# Perspective: Assuring the Quality of Protein in Infant Formula

**DOI:** 10.1016/j.advnut.2023.04.008

**Published:** 2023-04-25

**Authors:** John C. Wallingford

**Affiliations:** Nutrispectives, Spokane, WA, United States

**Keywords:** infant formula, protein quality, infant nutrition, human milk, protein efficiency ratio

## Abstract

Current regulations require that the assessment of protein quality in infant formula be determined using the protein efficiency ratio (PER) rat bioassay where the growth of rats fed a test protein is compared with the growth of rats fed casein. This review cites authoritative body opinions that the PER is not a preferred method for scoring protein quality, particularly as applied to the infant formula. Methodological recommendations specified by FDA and recent guidance propose to control nonprotein dietary variables in the PER. In contrast, the essential amino acid pattern of human milk has been adopted internationally as the standard for protein quality in infant formula. Because casein, the control protein in the PER fails to meet the standard of human milk essential amino acids, the PER based on casein can generate a false assurance of the quality of protein in an infant formula. FDA should revise the method of demonstrating the quality factor for the biological quality of protein to the essential amino acid pattern of human milk, which would be simpler, conform to international standards, and should be considered by FDA under a new statute. Alternate methods of determination of protein quality can be used selectively when there are questions about the digestibility of new protein sources or the effects of manufacturing processes.

**Statement of Significance**

This perspective identifies scientific and regulatory weaknesses in FDA’s protein quality evaluation of infant formula and proposes ways to update the regulations to reflect current scientific knowledge.

## Introduction

Section b(1) of the Infant Formula Act^1^ requires the Secretary to establish “quality factors for infant formulas to the extent possible consistent with current scientific knowledge.” Two quality factors were established in the 2014 regulation, 1 for normal physical growth, generally by a “growth monitoring study” and 1 for the quality of protein in formula using the protein efficiency ratio (PER)^2^. Only the United States, Canada^3^, and Mexico^4^ use PER to assess protein quality in infant formula. The PER measures the weight gain and food intake of rats fed a limiting amount of protein and the AOAC specifies the use of a reference group fed casein. Growth of rats fed test proteins and control rats fed casein is normalized to a standard casein value of 2.5 g of weight per gram gained per gram of protein consumed. The International Codex Standard for Infant Formula adopted in 1981 used the PER method for protein quality, but Codex abandoned PER in the 2007 revisions^5^. Other International bodies (EU^6^ and Food Safety Authority of Australia and New Zealand^7^) and individual countries^8^ adopted the amino acid pattern of human milk as their measure of protein quality for infant formula more than a decade ago. PER makes no attempt to assess nonprotein synthesis use of amino acids; such functions are presumably supported by the amino acid concentrations in human milk.

In its 2014 interim final rule^9^, FDA established the PER test as a regulatory requirement. The 2 main factors that determine protein quality are the amino acid composition of the protein and its digestibility. FDA selected the PER because “FDA is not aware^10^ of any other available method to assess protein bioavailability” (p. 8022) and the PER was the “only method that accounts for digestibility and absorption in a living animal system” (p. 8023). FDA cited only 2 historic papers by Hegsted and Chang published in 1965 that criticized the PER [1,2] but not the deliberations and criticisms by experts and authoritative bodies about measures of protein quality published since [[3], [4], [5], [6], [7], [8], [9], [10]].

## Problems with the PER

Common criticisms of the PER are that the rat has different essential amino acid requirements than humans; the PER estimates requirements only for growth and not maintenance; the outcome measure of weight gain does not take body composition into account; PER over-estimates quality of high-quality proteins such as are used in infant formulas; the PER assesses quality only with respect to the first limiting amino acid; and has low accuracy and reproducibility. Recent research suggests another criticism: rats of the age used in the PER have already been weaned and preadapted to diets for 3–7 d and have experienced a dramatic shift in the intestinal microbiome that accompanies the introduction of solid foods, the weaning reaction [11]. Potential effects of protein components reaching the lower gastrointestinal tract during early life cannot be studied [12].

As applied to infant formula, the test diets used in the PER have practical limitations. Intestinal lactase decreases rapidly after weaning, and rats fed a high lactose diet typical in infant formula are in a severely impaired state of chronic lactose intolerance. Many dietary and nondietary factors can affect the casein control result, including the strain of rat, sex, body weight and the age of the rat; dietary factors include water, fat, lactose, protein concentration and recently to include sulfate in mineral salts (Table 1) [[12], [13], [14], [15], [16], [17], [18], [19], [20], [21], [22], [23], [24], [25], [26], [27]]. The nonprotein variables can have large effects [14,15,24,28]; the concentration of protein [2] and supplementation of control diets with sulfur-containing amino acids [18,21] also affects the control group PER dramatically. Even controlling for these variables, the control group fed casein has a larger interlaboratory variability than other similar bioassays even in controlled validation studies [29], which is more pronounced in open literature [20]. The PER value for control rats fed casein ranges from just above 2 to >4.5. To reduce the variability across independent studies, the casein control result is normalized to 2.5, and the PER for the test protein is reported as a ratio to the control as normalized.

**TABLE 1:** Reported values of protein efficiency ratio for casein control diets

References	Casein result (g weight gain/g protein consumed)^1^	Comment
Morrison and Campbell [28]	2.75 male	Sex difference can be substantial, but PER design used male rats
	2.37 female	
Keane et al. [14]	2.33 (0% water); 3.37 (20% water)	Moisture is now controlled in PER design
	0.84 (50% water)	
Jansen [15]	3.24, 2.97 (Sprague Dawley)	Within-strain variability is less than between strain variability. Rat strain is now controlled in PER tests
	3.04, 2.64, 2.68 (Wister)	
	2.65, 2.77 (Sherman)	
	2.20 (Long–Evans)	
Hegsted and Chang [2]	1.79 [6.08]^2^; 2.80 [8.69]; 2.97 [12.96]; 3.0 [15.64]; 2.71 [19.11]; 2.19 [26.06]	Protein concentration, which is not specified as a single value per AOAC, is a critical variable. Most PER tests use 10% (w/w) protein
Harkins and Sarett [16]	2.89 ± 0.46^3^ (*n* = 10), 10% protein	Normal control values have a CV is 16%
Hackler et al. [17]	2.86 ± 0.28^3^ (*n =* 70), 10% protein	Comparative study from 7 laboratories shows pooled interlaboratory CV of 10%
McLaughlin et al. [18]	4.60 ± 0.42^3^ (*n =* 6), 8% protein with supplemental methionine, the limiting amino acid compared with rat requirements	Methionine supplementation dramatically improves control PER. This is because rat sulfur amino acid requirements are much greater than the amounts provided in casein
Sarwar et al. [19]	3.35 ± 0.07^4^ (*n =* 6 for each mean); 2.86 ± 0.09; 3.04 ± 0.07; 2.91 ± 0.10; 3.36 ± 0.10; 3.27 ± 0.16, 8% protein	Interlaboratory variability from 6 laboratories is substantial; compared with another interlaboratory study by Hackler et al. [17] that used 10% protein
Sarwar et al. [20]	3.56 ± 0.07^4^ (*n =* 10) 8% protein	Normal controls have wide variation
Sarwar [9]	3.85 (*n =* 8) 9% protein	Casein + methionine
Sarwar et al. [21]	4.73 ± 0.05^4^ (*n =* 8) 8% protein	Casein + methionine
Sarwar and Botting [22]	3.81 ± 0.15^4^ (*n =* 8) 8% protein	These studies are all from 1 laboratory using a standard casein
Mitchell et al. [23]	2.9 ± 0.05^4^ (*n =* 10), 10% protein	Normal controls reported with apparent low variability
Mitchell and Jenkins [24]	3.2 ± 0.13^4^ (*n =* 10), 10% protein	Unmodified casein control Control matched to soy formula #1 Control matched to soy formula #2 Control matched to milk formula #1 Control matched to milk formula #2 The authors state that lactose added to control diets for milk formulas is the likely cause of low PER
	3.1 ± 0.19^4^	
	3.2 ± 0.25^4^	
	2.3 ± 0.24^4^	
	2.1 ± 0.16^4^	
Forsum [25]	3.33 ± 0.30^3^ (*n =* 10), 10% protein	Normal control, higher than most reported values
Hernández et al. [26]	2.62 ± 0.04^4^ (*n =* 6), 10% protein	Normal control, in line with most reports
Babji et al. [27]	3.22; 2.30; 2.31; 2.93; 2.7	Results from 5 separate PER studies from the same laboratory. Within- laboratory variability is high (only mean values were reported)
Hoskin [12]	2.31	A significant improvement in the control PER result was reported as related to mineral sulfates
Product Safety Laboratories, 2022^5^	2.53 ± 0.37^3^ (*n =* 48 tests, 10 rats per test)	Accumulated results from 48 studies on infant formula and similar products in which fat, lactose, and moisture were controlled, protein at 10%. The CV is 15%

Abbreviations: PER, protein efficiency ratio.

1 Animal Nutrition Research Council (ANRC) reference casein, specified in the AOAC method, is no longer available, so the source and preparation of casein can be added to the list of dietary variables.

2 % protein in brackets.

3 SD.

4 SEM.

5 Personal communication, Product Safety Laboratories to JC Wallingford, December 2022.

Because casein is used as the control in every study, the variability around the casein control is not a reflection of the quality of protein in casein. Interlaboratory validation studies and studies reported from 1 laboratory also presumably control processing; in these reports, the variability in the casein control represents nonprotein-quality variability. If it is assumed that similar nonprotein-quality variability exists in the test formulas, there is a very high chance the PER will generate false-positive (suggesting the test protein is superior to casein when it is not) and false-negative results (suggesting the test protein is inferior to casein when it is not) (Supplemental Table 1).

In its rulemaking, FDA recognized that the official AOAC PER method as published was ill suited for infant formula, and required PER diets to be “appropriately modified,” naming adjustments in lactose, moisture, and dietary fat; the presumption was that greater control over the composition of the diets could address the limitations of the PER^11^. More recently, FDA recommended that the concentration of each vitamin, mineral, and optionally added substance be not >20% different between test and control diets^12^, essentially treating every nutrient as a potential confounder, even though not all nutrient deficiencies manifest as a reduction in rat growth [30]. FDA published draft guidance on the PER study design in February 2023^13^ requesting comments on numerous dietary variables of test and control diets, including the quantity and quality of fat in the diets, the minimum ratio of vitamin E to polyunsaturated fatty acids, the need to match nonlactose carbohydrates, the moisture content, the matching of minerals and whether ash was adequate or matching of individual minerals assayed by ICP-MS was needed, the matching of vitamins, the matching of fiber, the matching of inorganic sulfate, and the use of a second casein reference group with ratio of methionine to cysteine matched to the test diet. None of these recommendations has been shown to have an effect on the reliability of the assay, but they do make the PER quite complex to conduct.

## About the Quality of Casein

Although casein is the control for PER studies and is quantitatively important in cow milk, casein comprises only ∼15% of the protein in human milk [31]. The concentrations of 4 essential amino acids in bovine casein (mg/g N) are lower than their respective concentrations in human milk (Table 2) [32,33]. Expressed as mg/protein, casein has only 80% of the total sulfur amino acids as human milk; tryptophan in casein is only 72% of the human milk concentration; threonine is 95%; and leucine is only 54% of the concentration in human milk. A formula that has the exact same PER as bovine casein could be insufficient to satisfy the essential amino acid provided by human milk.

**TABLE 2:** Essential amino acids in bovine casein and human milk

Source	Amino acid (mg/g protein)										
	His	Ile	Leu	Lys	SAA			AAA	Thr	Trp	Val
					Total	Met	Cys				
Bovine casein [32]	26	90	51	75	28	25	3	100	41	13	61
Human milk [33]	23	51	94	63	35	14	21	87	43	18	50
Ratio casein:human milk	1.13	1.76	0.54^1^	1.19	0.80^1^	1.78	0.14^1^	1.15	0.95^1^	0.72^1^	1.22

Abbreviations: AAA, total aromatic amino acid; Cys, cysteine; His, histidine; Ile, isoleucine; Leu, leucine; Lys, lysine; Met, methionine; SAA, total sulfur-containing amino acids; Thr, threonine; Trp, tryptophan; Val, valine.

1 Numbers show the essential amino acids that are lower on an mg/g protein basis in casein than in human milk.

There is also a major difference in the content of sulfur-containing amino acids. The methionine:cysteine ratio in casein is >8:1. In contrast, cysteine in human milk exceeds the methionine concentration. Cysteine is disproportionally taken up in the intestine [34] and supplemental cysteine shifts methionine metabolism from trans-sulfuration to transmethylation [35]. Approximately 60% of the sulfur amino acid requirements can be satisfied by cysteine directly [35]. Cysteine also is a precursor to taurine, the most abundant free amino acid in human milk. Taurine is used in neonatal life to conjugate bile acids. Many infant formulas are supplemented with taurine, although it is not required as a nutrient in infant formula, nor is there a DRI for taurine, because adequate dietary cysteine supports adequate taurine biosynthesis. Approximately 25% of dietary cysteine is used directly for the biosynthesis of glutathione and mucins [34]. Dietary supplementation with cysteine and threonine (another amino acid for which casein fails to satisfy the human milk concentration) increases synthesis of intestinal mucins in rat compared with casein alone [36]. This suggests that the dietary supply of cysteine from casein may be sufficient for rat growth and glutathione biosynthesis, but not for optimal intestinal mucin production or production of taurine.

Similar arguments may be made for tryptophan. Tryptophan is not used only for protein synthesis, but it is also a precursor to niacin and serotonin. The PER study design cannot evaluate the flux from tryptophan to niacin because the PER diets are amply fortified with niacin itself; the PER is also incapable of evaluating the utilization of tryptophan to form serotonin, which may be impaired in diets that provide only casein-like concentrations of tryptophan. More recently, it has been learned that the metabolic intermediate on the tryptophan-to-niacin pathway, kynurenic acid, is a key stimulant for the production of regulatory T cells in the infant intestine, central to the development of tolerance [37]. Diets that match casein in tryptophan and yet fail to meet human milk tryptophan concentrations could generate acceptable rat growth but alter human immune system development.

A major clinical trial reported a transient deficit in weight gain of infants fed a hydrolyzed casein formula compared with infants fed standard intact protein whey hydrolysate formulas or breastfed infants [38], a finding replicated by a second group [39]. Leucine, a major regulator of protein synthesis [40], has only half the concentration in casein as it has in human milk (Table 2). Total protein in the hydrolysate formula was about twice that of age-matched human milk; therefore, the leucine supplied was probably close to the human milk reference. Might that transient deficit in weight gain have been caused by inadequate leucine at the age when protein requirements are greatest? It would be interesting to learn whether the deficit in the growth of infants fed casein hydrolysate formulas was in LBM. Using the amino acid pattern in human milk as a measure of protein quality presumably supports all these nongrowth functions of amino acids in infants. A PER test on human milk could show how much the “adequate” casein control differs from the quality of human milk.

Whey protein increases the dietary intake of amino acids that are the most limiting in casein. Formula manufacturers have long known that the combination of bovine whey protein and whole milk protein gives higher protein quality than casein; the blend of casein and whey most closely approximates the human milk amino acid pattern when whey comprises 70% of the protein^14^, ^15^.

FDA allows proteins with lower protein quality than casein to be used in infant formula, so long as there is an increase in the total protein concentration (21 CFR 107.100). The regulations allow protein with a PER value of 70% of casein to be used, provided the quantity is raised in reciprocal to their PER. When a protein source has deficiencies in essential amino acids, it is necessary to compensate by adding the deficient amino acids or by increasing the total amount of protein; the human milk essential amino acid pattern is a better reference than the PER when determining the concentration of total protein needed to provide the limiting amino acids.

## Alternate Methods of Establishing Protein Quality

Manufacturers of infant formula may be exempted from using the PER if an alternative method to the PER, based on sound scientific principles, is available (21 CFR 106.96(f)(3)). An FAO expert panel was convened in 1989 expressly to evaluate alternate methods to the PER for determination of protein quality. The report [4] addressed the infant formula specifically, “Since 1919, the PER method, which measures the ability of a protein to support growth in young, rapidly growing rats, has been used in many countries because it was believed to be the best predictor of clinical tests. However, after decades of use, it is now known that PER overestimates the value of some animal proteins for human growth while underestimating the value of some vegetable proteins for that purpose.” The body concluded, “The protein digestibility-corrected amino acid score is considered the most suitable regulatory method for evaluating protein quality of foods *and infant formulas*” (p. 42, emphasis added) and “The amino acid composition of human milk should be the basis of the scoring pattern to evaluate protein quality in foods for infants under 1 year of age.” In 2007, WHO reiterated that infant amino acid requirements were the appropriate reference when scoring protein digestibility-corrected amino acid score (PDCAAS) for infant foods [8].

In 2011, after another conference on assessing protein quality, FAO wrote, “…it was generally agreed that the PER method should be replaced by a more precise and appropriate method…, a method based on comparison of the amino acid content of food with human amino acid requirements (amino acid scoring system) was accepted as the most suitable approach for assessing the protein quality of foods.”^16^

The 2 foremost alternative methods using animal models and thereby assessing in vivo digestibility are the PDCAAS and the digestible indispensable amino acid score (DIAAS). PDCAAS, identified by FAO in 1989 as the preferred method [4], determines the digestibility of a protein in a rat bioassay, and then adjusts the amino acid composition of the test protein for its digestibility. PDCAAS is recognized by National Academy of Sciences [41] and American Academy of Pediatrics [42]; FDA adopted PDCAAS in 1991 for assessment of the quality of protein in all foods except foods for infants and young children^17^. PDCAAS has limitations [9,10], mainly, that quality relates to only the first limiting amino acid, and that bacterial metabolism of amino acids in the colon results in an overestimate of absorbed amino acids.

DIAAS, recommended for further study by WHO in 2007 [8], directly measures the digestion of each amino acid at the ileum, avoiding errors introduced by post-ileal metabolism. Its main drawback is that it is quite technical to perform and is not widely available. Rutherfurd and Moughan [43] used DIAAS to demonstrate that many milk protein preparations (lactic casein, sodium casein, calcium casein, whey protein concentrate, milk protein concentrate, α-lactalbumin) are highly digestible in rat. All essential amino acids in each milk protein preparation had ileal true digestibility >90%. In a separate study, Rutherfurd et al. [44] compared rat fecal digestibility to ileal digestibility of 14 food protein sources and found that the fecal digestibility gave higher values than ileal digestibility, especially for proteins that were not highly digestible. True ileal digestibility for all amino acids was 100% for whey protein isolate, 98% for whey protein concentrate, and 94% for milk protein isolate. Data from studies using DIAAS in pigs (Table 3) confirm the high digestibility of milk proteins used in infant formula [45]. The DIAAS score for amino acids from cow milk–based infant formula fed to piglets, which readily tolerate the high lactose food, averaged 96% [46], consistent with the DIAAS for cow milk protein preparations. DIAAS has much smaller errors around mean digestibility than PER has for growth of control rats. Validation studies of DIAAS in pigs showed between laboratory CV for indispensable amino acids of 1.8% [47]. DIAAS studies in infants have not been reported, but there are strong correlations between DIAAS scores of piglets and adult humans fed meals [48] and diets [49], with a pigs absorbing a few percent more amino acids than humans (the species difference should be taken into account when estimating protein quality for infants).

**TABLE 3:** Ileal digestibility of amino acids in proteins used in infant formula and wheat^1^

Indispensable amino acid	Ingredient					
	Digestibility %					
	WPI	WPC	MPC	SMP	SPI	Wheat^2^
Isoleucine	98	97	93	89	95	86
Leucine	99	98	98	94	95	86
Lysine	98	96	96	95	97	77
Methionine	98	97	97	96	96	88
Phenylalanine	98	96	97	94	96	87
Threonine	94	91	93	82	92	80
Tryptophan	100	98	97	91	96	74
Valine	97	95	94	90	94	83

Abbreviations: MPC, milk protein concentrate; SMP, skim milk powder; SPI, soy protein isolate; WPC, whey protein concentrate; WPI, whey protein isolate.

1 From Mathai et al. [45].

2 Shown for comparison with a protein source not used in infant formula.

Both PDCAAS and DIAAS reduce to the amino acid pattern of human milk when digestibility is 100%. Evidence from DIAAS studies is that amino acids from milk proteins have very high digestibility; little or no correction is needed to adjust the amount of the amino acid for its digestibility when milk proteins are used.

Digestibility of amino acids in dried foods is minimally affected by adducts formed during processing, for example, lysine condensation with lactose via the Maillard reaction. Because adducts may be absorbed but are not available for protein synthesis, DIAAS could overestimate the bioavailability of some amino acids. However, adduct formation accounts for only a few percent of total lysine in powdered infant formulas [50]. Processing of liquid formulas affects protein quality more than processing of powders [21,22,51], underscoring the need to evaluate protein quality in the infant formula as processed no matter the assay^18^.

## Moving toward Higher Protein Quality

The trend in infant formula composition is toward lower total protein amounts, which emphasizes the importance of protein quality. Higher than necessary protein intake during infancy may increase risk of later life obesity [52]; evidence of high ileal digestibility of cow milk proteins means that the “margin of safety” built into the protein requirement for infant formula to compensate for perceived reduced digestibility [42,53] no longer applies to cow milk proteins.

Given that the digestibility of cow milk proteins used in infant formula has been answered by methods that have superior precision compared with PER and establish the bioavailability of individual amino acids, there is little value in additional PER studies on milk proteins, which, as noted above, risk misleading results. For proteins with established bioavailability, human milk amino acid scoring is sufficient to ensure protein quality. For proteins for which digestion is not known, in vitro methods that correlate well to in vivo methods may be useful. These approaches remove the need to kill research animals. For novel proteins whose quality is not assured by in vitro tests, DIAAS is the optimal method to date.

Except for the addition of a selenium requirement^19^, the nutrient specifications in United States infant formulas have not been updated since 1980. New federal legislation requires FDA to consider where United States nutrient requirements for infant formula differ from those in Europe (and European requirements are close to those in the Codex Standard) and to assess the nutrient composition requirements every 4 y. Protein quantity and quality relative to casein are specified in United States regulations. The FDA should use this new mandate to revisit the method of demonstrating the quality factor for protein as well and remove the provision to compensate for quality less than casein by higher protein quantity.

Adopting the human milk amino acid pattern may require current American infant formula products to be reformulated. That happened already in markets in most of the world after the Codex adopted the human milk amino acid pattern as its standard for protein quality. Manufacturers may not advocate for alternatives to PER, as burdensome as the PER assay is, because it is a barrier to manufacturers seeking entry to the United States market. FDA has reinforced the barrier by recommending comprehensive matching of test and control formula nutrient composition. FDA may also be reluctant to revisit PER as its method of choice given the long use of PER in infant formula and recent guidance elaborating recommendations for the PER. However, the new law^20^ requires FDA to reconsider international standards and recommendations of authoritative science bodies and gives FDA reason to adopt alternative methods to the PER for establishing protein quality.

In conclusion, PER was adopted as the method of demonstrating the quality of protein for infant formula because it included the process of digestion. Historic data for the casein control group show great variability that can lead to false conclusions of infant formula protein quality, and preclude detection of small differences in protein quality. New guidance focuses on matching control and test diets for nonprotein dietary variables, but the recommendations have not been supported with evidence of improved precision or accuracy of the PER assay. Most importantly, because the amino acid pattern of bovine casein does not satisfy the human milk amino acid pattern, a positive PER test (that is, indicating adequate protein quality) could satisfy regulatory requirements without providing human milk concentrations of essential amino acids, which have important biologic functions other than growth. Current regulations that allow formulas that have quality even lower than casein to be commercialized, if compensated by greater quantity, should be based on the human milk amino acid pattern instead of PER. FDA should^21^ adopt amino acid pattern of human milk for proteins as the quality standard and recommend DIAAS for infant formulas with novel protein sources or processing. This approach satisfies the statutory requirement^22^ to “establish quality factors to the extent possible consistent with current scientific knowledge.”

## Funding

The author reported no funding received for this study.

## Author disclosures

The author reports no conflicts of interest.

## Footnotes

1 www.govinfo.gov/content/pkg/STATUTE-94/pdf/STATUTE-94-Pg1190.pdf

2 PER is specified in 21 CFR 106.96(f)) for infant formulas and in 101.9(c)(7) and foods intended for use by infants up to 12 mo of age, referencing AOAC Official Method 960.48 Protein Efficiency Ratio Rat Bioassay.

3 Canada tests infant formula protein quality after fat has been extracted.

4 https://extranet.who.int/nutrition/gina/sites/default/filesstore/MEX%202012%201-Norma%20Oficial%20Mexicana%20NOM-121-SSA1-2012_0.pdf

5 Adopted 2008, https://www.fao.org/fao-who-codexalimentarius/sh-proxy/en/?lnk=1&url=https%253A%252F%252Fworkspace.fao.org%252Fsites%252Fcodex%252FStandards%252FCXS%2B72-1981%252FCXS_072e.pdf; also noteworthy, Codex specified that to be used in infant formula, the PER result had to be at least 85% of the casein value, whereas in the US 21 CFR 107.100 allows protein to be used that has only 70% of the casein value.

6 Adopted 2007, https://eur-lex.europa.eu/legal-content/EN/TXT/PDF/?uri=CELEX:32006L0141&from=EN

7 Food Safety Authority of Australia and New Zealand, standard 2.9.1 https://www.legislation.gov.au/Details/F2022C00239, schedule 29-6

8 China: GB 10765, Chile article 493 follows Codex; RDC 43 for Brazil follows Codex; Malaysia 389

9 79 Federal Register, 7934, 10 February, 2014

10 This is an administrative procedure error. There is ample evidence that the agency was aware of discussions in the scientific community on the best methods for the determination of protein quality. CFSAN staff participated in some of these conferences by FAO and WHO, for example, the 1989 conference at NIH, Protein Quality Evaluation (4). CFSAN leads the US delegation to the Codex Committee on Nutrition and Foods for Special Dietary Uses, which the US co-chairs.

11 “Although the method has limitations with respect to assessment of the quality of protein sources for infant formulas, the limitations are greatly reduced by modification of the test and control diets” (79 Federal Register, 7934, 10 February, 2014, p. 8023)

12 https://www.fda.gov/food/workshops-meetings-webinars-food-and-dietary-supplements/webinar-series-discuss-infant-formula-transition-plan-exercise-enforcement-discretion-11172022

13 https://www.fda.gov/regulatory-information/search-fda-guidance-documents/draft-guidance-industry-protein-efficiency-ratio-rat-bioassay-studies-demonstrate-new-infant-formula

14 Wallingford, unpublished

15 FDA might have discussed whey-to-casein ratios as a protein quality factor. China revised national regulations in 2011 to specify a minimum content of 60% protein be from whey, the first instance of whey content as a protein quality factor.

16 FAO Expert consultation. Dietary protein quality evaluation in human nutrition. FAO Food Nutr. Pap. 2011;92:1–66.

17 21 CFR 101.9(c)(7)

18 21 CFR 106.96(c)(2)(iii)

19 https://www.federalregister.gov/documents/2015/06/23/2015-15394/infant-formula-the-addition-of-minimum-and-maximum-levels-of-selenium-to-infant-formula-and-related

20 www.congress.gov/117/bills/hr2617/BILLS-117hr2617enr.pdf, subpart D

21 Secretary shall consider any new scientific data or information related to infant formula nutrients, including international infant formula standards

22 https://www.govinfo.gov/content/pkg/STATUTE-100/pdf/STATUTE-100-Pg3207.pdf
