# Supplementary material for: Perspective: Assuring the Quality of Protein in Infant Formula
Source: Adv Nutr. 2023 Apr 25;14(4):585–91. doi: 10.1016/j.advnut.2023.04.008 (PMC10334145; doi:10.1016/j.advnut.2023.04.008)
Supplement: Multimedia component 1 [file mmc1.docx]

Perspective: Assuring the Quality of Protein in Infant Formula, Wallingford

Supplemental Table 1.

|  |  |  |  |  |  |  |  |  |
| --- | --- | --- | --- | --- | --- | --- | --- | --- |
|  | Estimating the chance of false positives and false negatives | | | | | |  |  |
|  |  |  |  |  |  |  |  |  |
|  |  |  |  | test protein result | |  |  |  |
|  |  |  | Below -1 SD | -1 to -0.5 SD | -0.5 to 0 SD | 0 to 0.5 SD | 0.5 to 1 SD | Above + 1 SD |
|  |  | probability | 0.16 | 0.15 | 0.19 | 0.19 | 0.15 | 0.16 |
|  | Below -1 SD | 0.16 | 0.0256 | 0.0240 | 0.0306 | 0.0306 | 0.0240 | 0.0256 |
| casein result | -1 to -0.5 SD | 0.15 | 0.0240 | 0.0225 | 0.0287 | 0.0287 | 0.0225 | 0.0240 |
|  | -0.5 to 0 SD | 0.19 | 0.0306 | 0.0287 | 0.0365 | 0.0365 | 0.0287 | 0.0306 |
|  | 0 to 0.5 SD | 0.19 | 0.0306 | 0.0287 | 0.0365 | 0.0365 | 0.0287 | 0.0306 |
|  | 0.5 to 1 SD | 0.15 | 0.0240 | 0.0225 | 0.0287 | 0.0287 | 0.0225 | 0.0240 |
|  | Above + 1 SD | 0.16 | 0.0256 | 0.0240 | 0.0306 | 0.0306 | 0.0240 | 0.0256 |

Legend for additional information

The table shows probabilities of results for test protein (columns) and casein controls (rows), based on casein control SD. The probabilities for both test and control results are shown in 0.5 SD increments. For example, the chance that the test protein result falls between 0 and +0.5 SD is 0.19.

a. area shaded red represents results when the casein control is 1 SD below the mean, i.e. below 2.16, Experience with FDA review is that when casein results are this low, the study is deemed not valid (see Hoskin, 2022). Thus 16% of tests are rejected, regardless of the outcome for the test group.

b. the blue cells are where the test protein PER is greater than control by 0.5 SD or more. Because the true protein quality of the test protein is equal to casein, these cells represent false positives (16% of outcomes), meaning they indicate the test protein is superior to casein when it is in fact not.

c. the yellow cells are where the test protein PER is less than control by 0.5 SD or more. Because the true protein quality of the test protein is equal to casein, these cells represent false negatives (27% of outcomes), meaning they indicate the test protein is inferior to casein when it is in fact not.

Assumptions are as follows.

1. The mean (2.53) and standard deviation (0.37) for casein controls reported by PSL are reasonable estimates. The PSL data represent by far the largest sample of tests (see Table 1) and the mean is close to the normalized mean for casein, 2.5.

2. The results are normally distributed. The SD of 0.37 means 68% of the control results fall between 2.16 and 2.9. 16% of results for control group are below 2.16. These have been considered to reflect a poorly controlled test, necessitating a repeat study (red cells).

3. Because casein is used as the control in every study, and because diets are compiled by PSL using standardized procedures, the variability around the casein control is not a reflection of the quality of casein in the diet as processed, it is non-protein-quality variability.

4. It is assumed that the test protein truly has the same protein quality as casein, and that variability around the result of a test protein is non-protein-quality variability, as it is for casein.

5. The difference between test and control that is statistically significant in 0.5 SD. This is arbitrary, but in line with reported data on PER studies.
